# Polyelectrolyte Coatings—A Viable Approach for Cultural Heritage Protection

**DOI:** 10.3390/ma16072873

**Published:** 2023-04-04

**Authors:** Ioana Cătălina Gîfu, Raluca Ianchiș, Cristina Lavinia Nistor, Cristian Petcu, Irina Fierascu, Radu Claudiu Fierascu

**Affiliations:** 1 Faculty of Chemical Engineering and Biotechnologies, University “Politehnica” of Bucharest, 060042 Bucharest, Romania; catalina.gifu@icechim.ro; 2 National Institute for Research & Development in Chemistry and Petrochemistry-ICECHIM–Bucharest, 060021 Bucharest, Romania; cristina.nistor@icechim.ro (C.L.N.); cristian.petcu@icechim.ro (C.P.); irina.fierascu@icechim.ro (I.F.); 3 Faculty of Horticulture, University of Agronomic Sciences and Veterinary Medicine of Bucharest, 011464 Bucharest, Romania

**Keywords:** polyelectrolyte, cultural heritage, layer-by-layer, preservation, protection, coatings

## Abstract

The continuous degradation of cultural heritage artifacts (due to different factors, including the rising air pollution, climate change or excessive biological activity, among others) requires the continuous development of protection strategies, technologies and materials. In this regard, polyelectrolytes have offered effective ways to fight against degradation but also to conserve the cultural heritage objects. In this review, we highlight the key developments in the creation and use of polyelectrolytes for the preservation, consolidation and cleaning of the cultural heritage artifacts (with particular focus on stone, metal and artifacts of organic nature, such as paper, leather, wood or textile). The state of the art in this area is presented, as well as future development perspectives.

## 1. Introduction

Cultural heritage includes three main categories, tangible (historic buildings, monuments, books, paintings and archaeological objects), intangible and natural heritage. The deterioration of cultural heritage over the past few decades has been attributed to rising air pollution, climate change and excessive biological activity. Cultural heritage degradation is an unfavorable process brought on by several elements such as humidity, pollution, heat and light that contribute to material degradation [1,2,3,4].

The humanity took advantage of polymers for a long time, but did not fully understand them until almost the end of World War II. Very few materials were accessible regarding the creation of the items required for life. For the majority of the construction, materials including steel, glass, wood, stone, brick and concrete were employed. Cotton, wood, jute and a few other agricultural items were used to make garments and other textiles. The introduction of new materials is determined by the sharp increase in demand for produced goods. These new materials refer to polymers and have a nearly unfathomable impact on how we live today. Epoxy glue, polymer paints, silicon hearts valves, polyethylene cups, fiberglass, nylon bearings, plastic bags, and Teflon-coated cookware are just a few examples of the usual products manufactured from polymers and the list practically never ends [5].

Both natural and synthetic polymers play a significant role in the comfort and ease of human life. They are in charge of sustaining life, as well as for things like food, transportation, communication, irrigation, containers, clothes, historical recording, structures and roadways. In this age, it is impossible to envision a human society without synthetic and organic polymers [6,7,8].

Science has a significant role in offering solutions to the pressing issues of food, clean, abundant water, air, energy and health in our rapidly technologically developing society. Understanding polymers and their related research studies can help us understand them better in our daily lives by giving us information and new perspectives. Understanding polymers could be made possible by the data gathered from the fundamental science courses. These data cover notions from science that are theoretical, factual and functional. It is helpful for individuals who wish to build a career in law, medical industries, business and other fields [5].

In recent decades, science provided innovative materials and methodologies for the examination, maintenance, and conservation of artwork, thus solving the problems of deterioration. However, the degradation of historical objects is an inevitable fact that requires the continuous development of advanced intelligent materials capable of counteracting the specific processes of degradation. Thus, an important challenge in research is the systemic formulation of new functional materials used to protect, conserve or restore art objects [9].

The most recent trends in the area of inorganic and organic materials for the conservation of different types of cultural heritage objects were recently reviewed by our group [10,11]. However, among the several polymer classes, the polyelectrolytes occupy a particular position. Polyelectrolytes are polymers possessing many ionizable groups. They could contain polycations or polyanions. When dissolved in polar solvents, polyelectrolytes are macromolecules that contain significant number of covalently bonded ionic groups. In general, different types of such groups may be present in polyelectrolytes. Solely one type of charged group, such as only carboxylate groups, involves homogeneous polyelectrolytes. We refer to a molecule as a polyampholyte when both positive (cationic) and negative (anionic) groups are present. Self-assembled structures, like linear protein assemblies or micelles, frequently have a lot of charged groups and may have characteristics that are very similar to those of polyelectrolytes. Developing on the self-assembly properties of polyelectrolytes, Decher et al. were the first scientists that developed the layer-by-layer (LbL) aggregation of electrolyte [12].

The main objective of the present review is to present the applicability of layer-over-layer deposited polyelectrolytes for the purpose of cultural heritage protection. The state of the art of polyelectrolyte coatings leading to multilayer structures and coatings will be presented first. We will pursue how several parameters influence the architecture of the resultant films and the self-assembly process, such as the type of polyelectrolytes and the sequence of assembly. Afterward, we report the recent improvements in the application of polyelectrolyte coatings for the protection, conservation or restoration of cultural heritage objects (with particular focus on stone, metal and artifacts of organic nature, such as paper, leather, wood or textile).

## 2. Recent Developments in Polyelectrolyte Coatings

**Figure 1:**
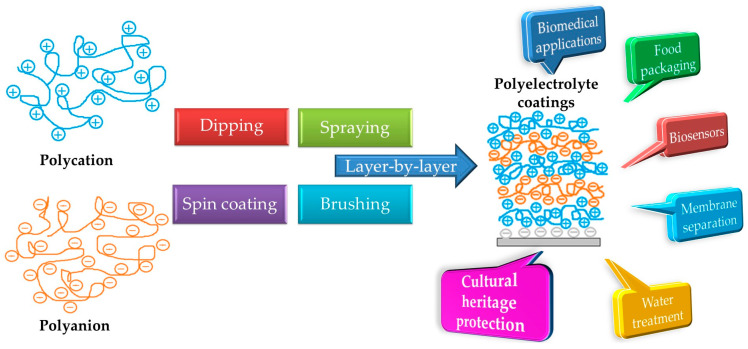
Applicative fields related to polyelectrolyte multilayered coatings.

Recent years have seen a focus on surface modification by polymers in an effort to expand the known adaptability of bulk polymeric materials to films and ultrathin coatings. In recent years, the self-organization of polyelectrolytes such as polysaccharides, heterocyclic aromatic compounds, clays, dyes, proteins/enzymes, carbon nanotubes, graphene oxide (GO) and other inorganics has been exploited more and more for the creation of well-defined surfaces and interfaces. With such techniques, multilayer coatings are formed spontaneously on substrates, due to the interactions between the substrate and polyelectrolyte [13]. By repeating the deposition process, uniform and homogeneous coatings can be obtained with a controlled thickness that varies from a few angstroms to a few microns [14]. The LbL architectures obtained from various natural and synthetic polyelectrolytes are very popular for the protection of different surfaces in many industrial fields, such as biomedicine, packaging, environmental, separation/purification membranes, catalysis, corrosion-resistant films, superhydrophobic, anti-fogging or anti-frosting surfaces, and conservation applications, among others (see Figure 1) [15,16,17,18,19].

It is notable that both weak and strong polyelectrolytes are used for the film build-up to achieve the ionic strength-dependent regulation of the characteristics of LbL assemblies. On the other hand, weak polyelectrolyte-based films are the only ones where pH has any effect on the LbL deposition process. Rubner and colleagues conducted a thorough investigation into the effect of pH on the development of LbL films made up of weak polyelectrolytes, using the polyelectrolyte pair PAH and poly(acrylic acid) (PAA) [20].

Polyelectrolyte multilayer films are used for biomedical applications such as biosensors, implantable materials, drug delivery, superhydrophobic surfaces, wound dressing, protein cell adhesion, mediation of cellular function and so on [15,21]. Natural biopolymers such as gelatin, chitosan (CH), carrageenan, alginate, collagen, fucoidan, hyaluran, polygalacturonic acid, heparin, chondroitin sulfate, among others, can be used as multilayer architecture [22,23]. Polysaccharides exhibit important properties for biomedical targeted applications, due to their stimuli responsiveness, mechanical toughness, low permeability, electrical conductivity, catalytic activity, biocompatibility, nontoxicity, bacteriostatic and antimicrobial properties. Coatings obtained from polysaccharides are one step closer to resembling the extracellular matrix and offer compositional individuality like inducing a particular cellular response. For instance, it is well known that hyaluronic acid interacts with a variety of receptors, including cell surface receptors [23]. CH can be used as an antimicrobial polyelectrolyte in combination with synthetic polyelectrolyte to obtain antimicrobial coatings for food packaging papers [16], while a poly(ethylene imine)-modified gelatin nanoparticle was utilized as a biodegradable and incredibly effective protein delivery technology for application in cancer treatment and regenerative medicine. [24,25,26].

Hyaluronic acid and alginate are the most researched polyanions, while CH and poly (L-lysine) are typically used as polycations. Polyelectrolyte multilayered coatings such as PLL/HA [27], CH/HA [28], and CH/ALG [29] have been extensively studied as substrates for cell adhesion. The CH/HEP and COL/HEP architectures were excellent candidates to be used as covering film to a variety of implants, including titanium implants for tissue engineering purposes, due to their blood-compatibility qualities [30]. Additionally, the CH/HA coatings showed outstanding adhesive and anti-inflammatory qualities that made them suitable for wound healing applications [31].

Synthetic polyelectrolytes, such as poly(sodium 4-styrene sulfonate) (PSS), (PAA), polymethyl methacrylate (PMMA), PAH, poly(diallyl dimethylammonium chloride) (PDADMAC), poly(ethylene imine) (PEI), poly(dimethylsiloxane) (PDMS), were also widely used in biomedical application. The advantage of using this type of polyelectrolyte is the facility of adjusting some parameters such as pH, ionic strength, thickness, ionic concentration and adhesion [15,32]. One of the most studied polyelectrolyte multilayers used in biomedical applications is PSS/PAH films due to their very good adhesion and proliferation of fibroblasts, endothelial and osteoblastic cells [33,34,35,36]. Feldötö et al. demonstrated that monoclonal mouse immunoglobulin G (IgG) could be immobilized on multilayers containing PAH and PSS. They observed that the binding capacity of immobilized IgG on PAH/PSS architecture is higher than on a clear surface [33]. An et al. reported a facile method for the construction of cross-linked polyelectrolyte multilayers using PSS/PAH by post-implantation and subsequent photochem [34]. PAH/PSS layer-by-layer assembly containing nanoporous silver submicrocubes were developed for electrochemical glucose sensing [35], while PEI/PAA polyelectrolyte multilayer architecture coated on PDMS conducted to long-term stability on the interface for protein detection [37].

Multilayer assemblies consisting of PDADMAC and PSS were obtained by Yu and al. to observe the influence of salt concentration on film growth. This LbL architecture can be applied as an advanced separation membrane [38]. César Vebber et al. produced thin films made of PAA, PAH, TiO_2_, CH and copper and evaluated their photoactivity, kinetics and recyclability for use in the photocatalytic destruction of EPs in water [39].

The polysaccharide-based film is extensively employed in agro-food industries to create edible films for food packaging. A variety of polysaccharide ingredients, including starch, CH, cellulose ethers, alginate, carrageenan and pectin, are used to create the polysaccharide-based film. These elements are created by plants. Edible film and coatings were created by the polymer chains of the polysaccharide-based components. Commercial uses for polysaccharides include thickening and gelling agents, crystallization inhibitors, stabilizers and encapsulating agents in the food industry [16,40,41].

When conventional restoration techniques were unable to increase the mechanical resistance of damaged artifacts, polymers were used in a few instances. Yet, the products used typically were commercial goods with qualities unsuited for the preservation of these artefacts [42,43]; often, polymers are used to prevent surface degradation [44]. As a result, modern materials based on acrylate polymers are frequently thought of when creating effective protective organic coatings for the preservation and protection of cultural heritage. [45,46,47,48]. The main benefits of protection coatings based on acrylates groups are their excellent mechanical resistance, good mechanical transparency, barrier qualities against oxygen, contaminants, moisture and UV light.

Moving away from synthetic polymers in favor of their naturally occurring equivalents is currently a significant trend, with consideration given to their eco-friendly, sustainable, non-toxic, and antibacterial qualities. Because they are reversible, degradable and allow retreatment, the characteristics of biodegradable polymers satisfy principles commonly recognized by the International Conservation Community [49]. Thus, biopolymers are considered good candidates for cultural heritage protection [50,51,52]. Moreover, the current literature indicates that the creation of novel, inventive materials is driven by efforts to reduce the environmental and ecological impact of materials. [53,54]. The fabrication of biopolymers using natural polymers and the manufacturing of biopolymer-based composites using solely natural additives have garnered a lot of attention during the past two decades. The performance and qualities of biomaterials (such as barrier properties, mechanical resistance, thermo-oxidation stability, long-term durability and photo-oxidation stability) must be equal to those of synthetic materials in order for them to replace polymers manufactured of fossil fuels. [51]. To achieve this, it is necessary to do research on and introduce naturally occurring additives that can improve the performance and properties of the biopolymers. [12,13,15]. CH, cellulose and hemicelluloses, pectin, starch, sodium alginate, polypeptides, etc. are excellent candidates for replacing numerous synthetic polymers [16]. Bio-polysaccharides are plentiful in nature, relatively inexpensive, non-toxic, biocompatible, renewable, and have also a reasonable film-forming ability [55].

## 3. Application of Polyelectrolyte Coatings for the Protection of Cultural Heritage Objects

**Figure 2:**
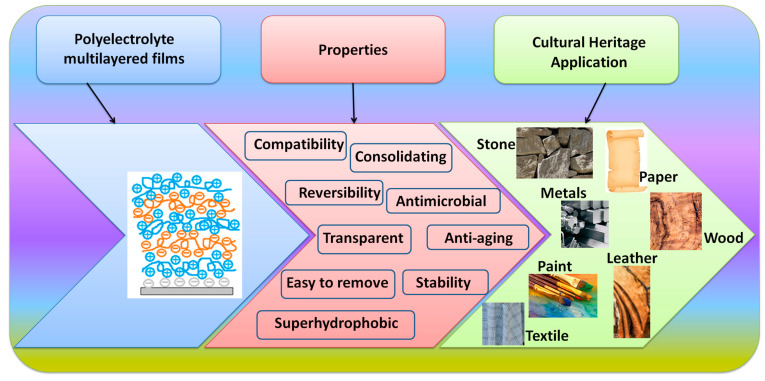
The use of polyelectrolyte multilayered coatings in cultural heritage application.

The creation of novel, tailored techniques that meet sustainability standards is the main problem facing researchers working on cultural heritage protection. Environmental protection, procedures that are non-destructive and/or reversible and restoration safety, as well as other ideas are included. Polyelectrolytes, due to their properties, can be used as coatings for artifacts such as those made of stone, metals, paper, textile, leather, etc. (Figure 2).

### 3.1. Coatings for Stone Artifacts

Stone artifacts are widely represented in the field of cultural heritage construction. The sculptures, monuments and buildings play a significant role in the materialization of the cultural heritage. The protection and preservation of stone artifacts are increasingly common in the field of cultural heritage [56,57,58,59,60,61,62]. Both indirect and direct conservation methods can be used on stone-based structures. In restoration and conservation procedures, surface coatings and consolidants are commonly used. The use of a variety of materials made of polymers, alkoxysilanes and nanocomposites such as biocides, water repellents and salt inhibitors, as well as consolidations to improve their mechanical characteristics, illustrates the general attributes of surface coverings. [63]. The mortars, cements and grouts are a further crucial component in the protection of cultural heritage stones [64], particularly when discussing buildings of cultural significance.

For the restoration to be successful, materials that are compatible with the stones must be developed [65,66,67]. Mortars can be developed with a variety of synthetic polymers, which improves the material’s mechanical, ion migration, acid attack, and freeze-thaw resistance qualities. The existence of several patents on this subject, some of which are decades old, supports the idea of a ready-to-market solution, which may be a useful tool for restorers. When different grades of limestone were impregnated, phase-change materials were created. These materials were then combined into mortars to produce materials with the right amount of workability, flexural strength, and compressive strength [59].

Due to their versatility, polyelectrolytes can be also used as protective coatings for stones [68]. Silica/polymer coating is a very good candidate for the protection of large-scale stone monuments because it is cheap and easy to apply [65]. Polyacrylate/silica hybrid represents a promising coating to be used as an anti-graffiti coating due to their ability to repels water and oil [69]. Hafez et al. present a protective coating using polyelectrolytes like PEI and PAA and hydroxyapatite particles by layer-by-layer technique [64]. Zárraga et al. [70] obtained a more elastic consolidant inside porous stones, by adding low amount of PDMS as an additive in TEOS- based stone consolidants. Moreover, PDMS was used as a polymeric adhesive to obtain uniform NP dispersion on the surface of the stone [6,66].

A coating developed for marble, consisting of photoactive, translucent and SiO_2_- TiO_2_ nanocomposites was created by Kapridaki et al. [71]. For various stone types, Kapridaki et al. has reported the development of novel hybrid nanomaterials based on different materials such as TiO_2_, TEOS and PDMS [72]. To conserve cultural heritage, La Russa et al. examined the self-cleaning capacity and hydrophobic properties of nano-TiO_2_ coatings using various binder materials [73]. The ideal amounts of TiO_2_ NPs to include in a nanocomposite coating of TiO_2_-SiO_2_-PDMS as a self-cleaning coating for stone materials have been discussed by Crupi et al. [74]. Protective coatings such as Ag-TiO_2_/PDMSfor Lecce stone have recently been studied by Ben Chobba et al. for their self-cleaning and antibacterial qualities [69], while La Russa et al. revealed that Paraloid B72 is indeed a poor adhesive to be combined with TiO_2_ nanoparticles since it causes a significant alteration of both stones surfaces and exhibits little hydrophobic and photo-degradative action [73].

Andreotti et al. developed stones treatment consisting of PAA, alginic acid (ALA), CH and tannic acid (TA) [67]. The detrimental effects of PAA were assessed after crystallization testing with uninterrupted capillary flow on a section of the stone artifacts, and the resistance of the stone was likewise rendered worse by ALA and CH [67]. Polymethyl methacrylate (PMMA) and perfluorinated polyether (PFPE) were the polymers used in polymer–silica nanoparticles composite films as protective coatings for stone-based monuments [65]. Ocak et al. [49] coated marble surfaces with films of biopolymers such as CH, zein, polyhydroxybutyrate and poly-lactide thus providing significant protection of up to 60%.

Sodium polyacrylates that have been chemically modified to have hydrophobic groups attached to their hydrophilic backbones are known as hydrophobically modified sodium polyacrylates (NaPACns) [75]. When electrostatic (repulsion and attraction) and hydrophobic interactions intersect, hydrophobically modified sodium polyacrylates exhibit unique features (viscoelastic and rheological properties, foam film drainage, stability, pH-responsive character, etc.) that makes them valuable in a range of applications (including, but not limited to paints industry, emulsifiers, environmental protection, biomedicine, etc.) [76]. The various sectors in which these materials can be used provide justification for their use and research. Early research showed that NaPACns had the ability to host hydrophobic molecules [77], as well as possess antibacterial properties, as demonstrated on *Staphylococcus aureus*, *Escherichia coli*, *Pseudomonas aeruginosa* and *Candida albicans*, using multilayer films obtained from HMPA using the LbL method [78]. The water-repellent properties [79], as well as their long-lasting effect [80], were also presented in published studies. Stone material protection coatings were created using sodium polyacrylate/oxide nanoparticle films that had been hydrophobized [81].

Several scientists working in stone conservation and preservation are now interested in antimcrobial and antifouling coatings [82,83,84,85]. The biodeteriogens, the artifact’s substance and its condition of conservation influence the cleaning method selection, however the application of chemicals (biocides) is the most popular antifouling technique. Natural goods antifouling are preferable to conventional hazardous biocides because they are less toxic, effective at low concentrations, biodegradable and have broad range antifouling activity. Common natural antifoulants include anesthetics, toxins, inhibitors, surface-active compounds and repellents. The majority of the natural antifouling substances that have been found so far include steroids, terpenoids, phenolics, carotenoids, alkaloids, furanones and peptides [82,86,87,88,89]. In order to develop an antifouling treatment with a higher efficacy over time, Ruggiero et al. suggested developing a novel coating with antifouling properties [90]. Liu et al. have prepered by liquid flame spray technique new polyimide–copper devices, with the polyimide shell encasing copper nanoparticles wich have anti-corrosion/fouling properties [91].

The usage of polyelectrolyte, and polymer-nanoparticle hybrids to enhance the performance of coatings for the protection of stone materials has been covered in this section. An overall improvement in the stone’s protection was noted in the scientific literature. The application of nanostructured coatings in cultural heritage conservation is fairly limited because there has not been a long-term investigation of the behavior of those materials. Nevertheless, such coatings were successfully synthesized and tested on simulated samples.

### 3.2. Coatings for Metal Artifacts

The metallic artifacts core material must be protected from patina which is caused by the interaction of water and oxygen in the environment. As it can protect the metal’s surface and removing it could damage the already-delicate object, it is now preferred to leave the natural patina on antique metals [55]. An appealing strategy for the creation of durable, sustainable and protective coatings is the use of biopolymer materials derived from renewable sources, which serve as a protective coating and storage capsule for corrosion inhibitors [68,92]. The use of hazardous solvents required for the use and elimination of often employed commercial protecting finishes made of benzotriazole spread in acrylic resins or microcrystalline waxes, may be avoided by using water soluble natural polymers. Therefore, the creation of novel water-soluble chemicals is essential, particularly for conservation procedures regarding immovable works of art. This information is crucial for enabling the stakeholders to agree that nanostructured coatings are appropriate for protecting constructed heritage, which needs to be preserved for long time.

CH has become a very interesting material and a good replacement for traditional coating systems used in the production of polymer-based coatings due to its inherent properties, such as antimicrobial activity, bio-compatibility and degradability, excellent adhesion to metal substrates, and the ability to reversibly form complexes with anti-corrosion properties [93]. Giuliani et al. [94] investigated the efficacy of coatings based on chitosan to prevent deterioration processes in copper alloy substrates for applications in cultural heritage preservation. A hybrid coating with the ability to preserve and inhibit corrosion for bronzes was made by using BTA and alginate [95]. Layer-by-layer constructed nano-reservoirs of corrosion inhibitor were incorporated by Zheludkevich et al. to obtain the protective coating’s intelligent, self-healing capability [96].

Polyelectrolytes are efficient for sealing surface defects and have excellent adherence to the substrate surface. Polyelectrolyte multilayers have sparked a lot of interest recently in terms of corrosion prevention [97]. Numerous reactions that affect the characteristics and composition of the metal surface, as well as the immediate surrounding environment, occur in conjunction with corrosion processes. Because the degree of dissociation of the polyelectrolytes is affected by the local pH value, polyelectrolyte films can change their chemical composition with pH. By fabricating the films at a pH regime where one of the weakly charged polymers and the other is substantially charged, the films can be enriched in one polymer relative to the other [98]. By using a scanning vibrating electrode technique that generates current density maps over a chosen area of the sample, Andreeva et al. investigated the anticorrosion activity of PEI, PAA, PSS and PDADMAC [99].

Other types of copolymers mixtures (not electrolytes by their strict definition) are commonly used as adhesives, consolidants and protective layers; this category includes commercial formulations, such as copolymer of methyl acrylate and ethyl methacrylate known as Paraloid^®^ B-72 used in conservation for restoration of inorganic and organic materials [100]. Methyl methacrylate and ethyl acrylate copolymer, a component of the Incralac^®^ coating formulation, is known commercially as Paraloid^®^ B-44. On the other hand, conservators use Zapon^®^ (Lechler, Italy), a lacquer that contains cellulose nitrate polymer that is soluble in several solvents (such as ethanol, ethyl acetate, and ethyl glycol) as a varnish, adhesive and consolidant [101]. Paraloid B67 and Paraloid B72 are other commercial acrylic polymers used as water repellents [102].

### 3.3. Coatings for Organic Artifacts (Paper, Leather, Wood, Textile)

Ancient and archeological textiles are priceless cultural assets that must be safeguarded from deterioration caused by environmental factors over time, such as changes in temperature, light, and humidity, as well as contaminants in the air. Usually, textiles with historically and aesthetic significance are preserved or restored using commercial products with attributes not specifically intended for the conservation of natural polymers. Numerous novel coatings made of nanostructured materials have been developed recently, specifically for the preservation and protection of cultural heritage [103,104].

Polymers previously used in the field of restoration and conservation of cultural heritage (water-based polyurethanes and fluorinated copolymers) were investigated as coatings for artifacts from the textile history [105]. Furthermore, fluoroacrylic co-polymer and polydimethylsiloxane can be applied both by spray and immersion, leading to water-repellent papers and textiles [106].

Books, documents, manuscripts and newspapers are only a few examples of historical paper collections that are vulnerable and quickly altered by human handling, biological pathogens and storage conditions (such as weather, microorganism activity and photo-oxidation). The mechanical properties of paper archives can be reduced by biodeterioration induced by fungi or bacteria, which results in irreversible deterioration and substantial loss of priceless data. While this is happening, excessive sun exposure would accelerate UVA and UVB radiation’s breakdown of cellulose molecules. Therefore, in order to delay the degradation in the maintenance of these paper collections, preventive conservation is required [41,107,108,109,110,111].

Jia et al. [107] present a straightforward and economical method for incorporating ZnO nanoparticles into cellulose nanocrystals (CNC) using in situ solution casting. The nanocomposites serve as consolidated medium and shield paper-based products from UV light, fungi and bacteria. Zhang et al. [112] investigated the photo-stability of a laminated assembly made of hydroxypropyl cellulose and 2, 4-dihydroxybenzophenone for usage as consolidating adhesives for the preservation of cultural artefacts. They demonstrated how some photodegradation inhibitors and popular polymers might prevent paper with various colors and pigment combinations from fading [112].

Superior durability may be seen in the CH nanoparticle-treated paper. Due to ionic interaction with free H^+^, protonated amines are present as ammonium salts, and the residual NH_2_ can be employed as a base. Jia et al. claim that four typical fungi found in libraries and museums are used to test the antibacterial effectiveness of paper covered with CH nanoparticles. The outcomes demonstrate the potent antibacterial activity of CH nanoparticles [108]. According to Totolin and colleagues, the plasma polymer P(MMA-co-EtA) film formed by low pressure non equilibrium plasma polymerization allows the creation of a “polymer-like” structure while maintaining the functions of the monomers [113]. The coatings made of poly(vinylidene fluoride/co-hexafluoropropylene)/1H, 1H, 2H, 2H-perfluorodecyltriethoxysilane exhibited high superamphiphobicity and very good stability against diverse severe treatments. Such a strong, superamphiphobic fiber coating may find applications in protection of ancient and archeological textiles [114].

## 4. Conclusions and Future Perspectives

Cultural heritage is a priceless socioeconomic asset. When protected and made accessible, works of art promote employment growth, tourism, social inclusion and cultural identity. Artifacts are unavoidably subject to periodic degradation processes, notwithstanding their varied origin and composition. Climate change, biocontamination, natural disasters (fires, floods), anthropogenic causes (vandalism, pollution, incorrect restoration interventions) and environmental factors (light, temperature, relative humidity) all pose risks to the heritage preservation and its transmission to future generations. It is now necessary to address these issues, and science has responded over the past few decades by developing novel tools and techniques.

One of the earliest restoration techniques is the safeguarding of artistic surfaces. Traditional varnish, nevertheless, age and they can even hasten the deterioration and modification of artworks, as was covered in the preceding sections. Over the past few decades, researchers developed several promising novel formulations in an effort to find substitute materials to shield surfaces from dirt, erosion and corrosion.

The development of novel materials for the conservation of artworks is a broad field with many unresolved issues and untapped potential. Despite the wide range of systems and applications covered in this analysis, more research has to be done. In order to comply with the EU Green Deal demands, one of the primary trends in conservation science is the expanding of green chemistry-based formulations. The use of natural materials as consolidants, gelling agents, or film-forming molecules is strongly recommended based on the same principles. In museums and storage facilities, polyelectrolytes compounds could be employed as coatings to protect artefacts through preventive conservation.

This review study provided an overview of many elements of using the polyelectrolytes as functional coatings for diverse applications. Natural or chemical polyelectrolyte materials are used in coating manufacture due to their ability to improve the physical-chemical characteristics of the products. Polyelectrolyte functionalized coatings are potential candidates in green chemistry context, where the use of substances with fewer negative effects is required.

The scope and variety of conservation challenges continue to be enormous. The preservation of outdoor artifacts, archaeological sites and historic architectures is under jeopardy due to the worrying issue of climate change and natural disasters. Nonetheless, industrial materials that are not designed to survive a long time have frequently been used to create contemporary and modern art, and they still are. Due to quick degradation processes, paint layers and plastic surfaces can show significant sensitivity to traditional solvents and cleaning agents or exhibit severe mechanical failure. Overall, materials scientists are still fully encouraged to develop new cutting-edge materials to protect our valuable cultural heritage.

## Data Availability

Not applicable.

## Abbreviations

**Abbreviation**	**Description**
ALA	Alginic acid
ALG	Alginate
CNC	Cellulose nanocrystals
CH	Chitosan
GO	Graphene oxide
HA	Hyaluronic acid
HEP	Heparin
HMPA	hydrophobically modified poly(acrylic acid)
NaPACns	Hydrophobically modified sodium polyacrylates
IgG	Monoclonal mouse immunoglobulin G
LBL	Layer-by-layer
PFPE	Perfluorinated polyether
PAA	Poly(acrylic acid)
PAH	Poly(allylamine hydrochloride)
PDADMAC	Poly(diallyl dimethylammonium chloride)
PDMS	Poly(dimethylsiloxane)
PEI	Poly(ethylene imine)
PLL	Poly(L-lysine)
PMMA	Poly(methyl methacrylate)
PSS	Poly(sodium 4-styrene sulfonate)
NPs	Nanoparticles
TA	Tannic acid

## References

[1] Bonazza A., Messina P., Sabbioni C., Grossi C.M., Brimblecombe P. (2009). Mapping the Impact of Climate Change on Surface Recession of Carbonate Buildings in Europe. Sci. Total Environ..

[2] Coelho G.B.A., Silva H.E., Henriques F.M.A. (2020). Impact of Climate Change in Cultural Heritage: From Energy Consumption to Artefacts’ Conservation and Building Rehabilitation. Energy Build..

[3] Turo F.D. (2016). Impacts of Air Pollution on Cultural Heritage Corrosion at European Level: What Has Been Achieved and What Are the Future Scenarios. Environ. Pollut..

[4] Willis K.G., Ginsburgh V.A., Throsby D. (2014). The Use of Stated Preference Methods to Value Cultural Heritage. Handbook of the Economics of Art and Culture.

[5] Gandini A., Belgacem M., Belgacem M., Gandini A. (2008). The state of the Art. Monomers, Polymers and Composites from Renewable Resources.

[6] Weththimuni M.L., Chobba M.B., Sacchi D., Messaoud M., Licchelli M. (2022). Durable Polymer Coatings: A Comparative Study of PDMS-Based Nanocomposites as Protective Coatings for Stone Materials. Chemistry.

[7] Sanchez-Salvador J.L., Balea A., Monte M.C., Negro C., Blanco A. (2021). Chitosan Grafted/Cross-Linked with Biodegradable Polymers: A Review. Int. J. Biol. Macromol..

[8] Begum S., Yuhana N.Y., Md Saleh N., Kamarudin N.H.N., Sulong A.B. (2021). Review of Chitosan Composite as a Heavy Metal Adsorbent: Material Preparation and Properties. Carbohydr. Polym..

[9] Baglioni M., Poggi G., Chelazzi D., Baglioni P. (2021). Advanced Materials in Cultural Heritage Conservation. Molecules.

[10] Fistos T., Fierascu I., Fierascu R.C. (2022). Recent Developments in the Application of Inorganic Nanomaterials and Nanosystems for the Protection of Cultural Heritage Organic Artifacts. Nanomaterials.

[11] Fistos T., Fierascu I., Doni M., Chican I.E., Fierascu R.C. (2022). A Short Overview of Recent Developments in the Application of Polymeric Materials for the Conservation of Stone Cultural Heritage Elements. Materials.

[12] Decher G. (1997). Fuzzy Nanoassemblies: Toward Layered Polymeric Multicomposites. Science.

[13] Bertrand P., Jonas A., Laschewsky A., Legras R. (2000). Ultrathin Polymer Coatings by Complexation of Polyelectrolytes at Interfaces: Suitable Materials, Structure and Properties. Macromol. Rapid Commun..

[14] Kötz J., Kosmella S., Beitz T. (2001). Self-Assembled Polyelectrolyte Systems. Prog. Polym. Sci..

[15] Costa R.R., Mano J.F. (2014). Polyelectrolyte Multilayered Assemblies in Biomedical Technologies. Chem. Soc. Rev..

[16] Nechita P., Roman (Iana-Roman) M. (2020). Review on Polysaccharides Used in Coatings for Food Packaging Papers. Coatings.

[17] Li Q., Wang S., Jin X., Huang C., Xiang Z. (2020). The Application of Polysaccharides and Their Derivatives in Pigment, Barrier, and Functional Paper Coatings. Polymers.

[18] De Carvalho M.A., Lazari-Carvalho P.C., Polonial I.F., de Souza J.B., Magne P. (2021). Significance of Immediate Dentin Sealing and Flowable Resin Coating Reinforcement for Unfilled/Lightly Filled Adhesive Systems. J. Esthet. Restor. Dent..

[19] Vergaro V., Scarlino F., Bellomo C., Rinaldi R., Vergara D., Maffia M., Baldassarre F., Giannelli G., Zhang X., Lvov Y.M. (2011). Drug-Loaded Polyelectrolyte Microcapsules for Sustained Targeting of Cancer Cells. Adv. Drug Deliv. Rev..

[20] Stockton W.B., Rubner M.F. (1997). Molecular-Level Processing of Conjugated Polymers. 4. Layer-by-Layer Manipulation of Polyaniline via Hydrogen-Bonding Interactions. Macromolecules.

[21] Piccinini E., Bliem C., Reiner-Rozman C., Battaglini F., Azzaroni O., Knoll W. (2017). Enzyme-Polyelectrolyte Multilayer Assemblies on Reduced Graphene Oxide Field-Effect Transistors for Biosensing Applications. Biosens. Bioelectron..

[22] Zhang J., Senger B., Vautier D., Picart C., Schaaf P., Voegel J.-C., Lavalle P. (2005). Natural Polyelectrolyte Films Based on Layer-by Layer Deposition of Collagen and Hyaluronic Acid. Biomaterials.

[23] Silva J.M., Reis R.L., Mano J.F. (2016). Biomimetic Extracellular Environment Based on Natural Origin Polyelectrolyte Multilayers. Small.

[24] Chou M.-J., Yu H.-Y., Hsia J.-C., Chen Y.-H., Hung T.-T., Chao H.-M., Chern E., Huang Y.-Y. (2018). Highly Efficient Intracellular Protein Delivery by Cationic Polyethyleneimine-Modified Gelatin Nanoparticles. Materials.

[25] Lin S.-F., Jiang P.-L., Tsai J.-S., Huang Y.-Y., Lin S.-Y., Lin J.-H., Liu D.-Z. (2019). Surface Assembly of Poly(I:C) on Polyethyleneimine-Modified Gelatin Nanoparticles as Immunostimulatory Carriers for Mucosal Antigen Delivery. J. Biomed. Mater. Res. B Appl. Biomater..

[26] Zwiorek K., Kloeckner J., Wagner E., Coester C. (2005). Gelatin Nanoparticles as a New and Simple Gene Delivery System. J. Pharm. Pharm. Sci..

[27] Crouzier T., Picart C. (2009). Ion Pairing and Hydration in Polyelectrolyte Multilayer Films Containing Polysaccharides. Biomacromolecules.

[28] Almodóvar J., Place L.W., Gogolski J., Erickson K., Kipper M.J. (2011). Layer-by-Layer Assembly of Polysaccharide-Based Polyelectrolyte Multilayers: A Spectroscopic Study of Hydrophilicity, Composition, and Ion Pairing. Biomacromolecules.

[29] Park K., Choi D., Hong J. (2018). Nanostructured Polymer Thin Films Fabricated with Brush-Based Layer-by-Layer Self-Assembly for Site-Selective Construction and Drug Release. Sci. Rep..

[30] Criado-Gonzalez M., Mijangos C., Hernández R. (2021). Polyelectrolyte Multilayer Films Based on Natural Polymers: From Fundamentals to Bio-Applications. Polymers.

[31] Richert L., Lavalle P., Payan E., Shu X.Z., Prestwich G.D., Stoltz J.-F., Schaaf P., Voegel J.-C., Picart C. (2004). Layer by Layer Buildup of Polysaccharide Films: Physical Chemistry and Cellular Adhesion Aspects. Langmuir.

[32] Gribova V., Auzely-Velty R., Picart C. (2012). Polyelectrolyte Multilayer Assemblies on Materials Surfaces: From Cell Adhesion to Tissue Engineering. Chem. Mater..

[33] Feldötö Z., Lundin M., Braesch-Andersen S., Blomberg E. (2011). Adsorption of IgG on/in a PAH/PSS Multilayer Film: Layer Structure and Cell Response. J. Colloid Interface Sci..

[34] An Q., Zhou Y., Zhang Y., Zhang Y., Shi F. (2014). A Facile Method for the Fabrication of Covalently Linked PAH/PSS Layer-by-Layer Films. RSC Adv..

[35] Viswanathan P., Kim Y.J., Hong J.D. (2020). Nanoporous Silver Submicrocubes Layer by Layer Encapsulated with Polyelectrolyte Films: Nonenzymatic Catalysis for Glucose Monitoring. Langmuir.

[36] Ahmad M., Yaroshchuk A., Bruening M.L. (2020). Moderate PH Changes Alter the Fluxes, Selectivities and Limiting Currents in Ion Transport through Polyelectrolyte Multilayers Deposited on Membranes. J. Membr. Sci..

[37] Khnouf R., Karasneh D., Albiss B.A. (2016). Protein Immobilization on the Surface of Polydimethylsiloxane and Polymethyl Methacrylate Microfluidic Devices. Electrophoresis.

[38] Yu L., Yuan W., Liu X., Xu X., Ruan S. (2017). Asymmetry of the Free-Standing Polyelectrolyte Multilayers. Appl. Surf. Sci..

[39] Vebber M.C., Aguzzoli C., Beltrami L.V.R., Fetter G., da Silva Crespo J., Giovanela M. (2019). Self-Assembled Thin Films of PAA/PAH/TiO2 for the Photooxidation of Ibuprofen. Part II: Characterization, Sensitization, Kinetics and Reutilization. Chem. Eng. J..

[40] Kumar N., Neeraj (2019). Polysaccharide-Based Component and Their Relevance in Edible Film/Coating: A Review. Nutr. Food Sci..

[41] Amini E., Azadfallah M., Layeghi M., Talaei-Hassanloui R. (2016). Silver-Nanoparticle-Impregnated Cellulose Nanofiber Coating for Packaging Paper. Cellulose.

[42] Nikaido T., Tagami J., Yatani H., Ohkubo C., Nihei T., Koizumi H., Maseki T., Nishiyama Y., Takigawa T., Tsubota Y. (2018). Concept and Clinical Application of the Resin-Coating Technique for Indirect Restorations. Dent. Mater. J..

[43] Rizzante F.A.P., Bombonatti J.S.F., Vasconcelos L., Porto T.S., Teich S., Mondelli R.F.L. (2019). Influence of Resin-Coating Agents on the Roughness and Color of Composite Resins. J. Prosthet. Dent..

[44] Novakovic D., Peltonen L., Isomäki A., Fraser-Miller S.J., Nielsen L.H., Laaksonen T., Strachan C.J. (2020). Surface Stabilization and Dissolution Rate Improvement of Amorphous Compacts with Thin Polymer Coatings: Can We Have It All?. Mol. Pharm..

[45] Carretti E., Chelazzi D., Rocchigiani G., Baglioni P., Poggi G., Dei L. (2013). Interactions between Nanostructured Calcium Hydroxide and Acrylate Copolymers: Implications in Cultural Heritage Conservation. Langmuir.

[46] Baglioni M., Montis C., Chelazzi D., Giorgi R., Berti D., Baglioni P. (2018). Polymer Film Dewetting by Water/Surfactant/Good-Solvent Mixtures: A Mechanistic Insight and Its Implications for the Conservation of Cultural Heritage. Angew. Chem..

[47] Castel A., Gutfreund P., Cabane B., Rharbi Y. (2020). Stability of Fluid Ultrathin Polymer Films in Contact with Solvent-Loaded Gels for Cultural Heritage. Langmuir.

[48] Baglioni M., Montis C., Brandi F., Guaragnone T., Meazzini I., Baglioni P., Berti D. (2017). Dewetting Acrylic Polymer Films with Water/Propylene Carbonate/Surfactant Mixtures—Implications for Cultural Heritage Conservation. Phys. Chem. Chem. Phys..

[49] Ocak Y., Sofuoglu A., Tihminlioglu F., Böke H. (2009). Protection of Marble Surfaces by Using Biodegradable Polymers as Coating Agent. Prog. Org. Coat..

[50] Infurna G., Cavallaro G., Lazzara G., Milioto S., Dintcheva N.T. (2020). Bionanocomposite Films Containing Halloysite Nanotubes and Natural Antioxidants with Enhanced Performance and Durability as Promising Materials for Cultural Heritage Protection. Polymers.

[51] Bertolino V., Cavallaro G., Milioto S., Lazzara G. (2020). Polysaccharides/Halloysite Nanotubes for Smart Bionanocomposite Materials. Carbohydr. Polym..

[52] Andreotti S., Franzoni E., Fabbri P. (2018). Poly(Hydroxyalkanoate)s-Based Hydrophobic Coatings for the Protection of Stone in Cultural Heritage. Materials.

[53] Kumar D., Gihar S., Shrivash M.K., Kumar P., Kundu P.P. (2020). A Review on the Synthesis of Graft Copolymers of Chitosan and Their Potential Applications. Int. J. Biol. Macromol..

[54] Valentini F., Carbone M., Palleschi G. (2013). Carbon Nanostructured Materials for Applications in Nano-Medicine, Cultural Heritage, and Electrochemical Biosensors. Anal. Bioanal. Chem..

[55] Hassan B., Chatha S.A.S., Hussain A.I., Zia K.M., Akhtar N. (2018). Recent Advances on Polysaccharides, Lipids and Protein Based Edible Films and Coatings: A Review. Int. J. Biol. Macromol..

[56] Cao Y., Salvini A., Camaiti M. (2017). Oligoamide Grafted with Perfluoropolyether Blocks: A Potential Protective Coating for Stone Materials. Prog. Org. Coat..

[57] Cao Y., Salvini A., Camaiti M. (2021). One-Step Fabrication of Robust and Durable Superamphiphobic, Self-Cleaning Surface for Outdoor and in Situ Application on Building Substrates. J. Colloid Interface Sci..

[58] Eyssautier S., Calandra I., Vaillant-Gaveau N., Fronteau G., Thomachot-Schneider C., Hubert J., Pleck J., Gommeaux M. (2018). A New Preventive Coating for Building Stones Mixing a Water Repellent and an Eco-Friendly Biocide. Prog. Org. Coat..

[59] Alvarez de Buergo M., Saladino M., Renda V., Caponetti E. (2020). Assessment of Protection Treatments for Carbonatic Stone Using Nanocomposite Coatings. Prog. Org. Coat..

[60] David M.E., Ion R.-M., Grigorescu R.M., Iancu L., Andrei E.R. (2020). Nanomaterials Used in Conservation and Restoration of Cultural Heritage: An Up-to-Date Overview. Materials.

[61] Lettieri M., Masieri M., Aquaro M., Dilorenzo D., Frigione M. (2021). Eco-Friendly Protective Coating to Extend the Life of Art-Works and Structures Made in Porous Stone Materials. Coatings.

[62] Ruffolo S.A., La Russa M.F. (2019). Nanostructured Coatings for Stone Protection: An Overview. Front. Mater..

[63] Tabasso M.L. (1995). Acrylic Polymers for the Conservation of Stone: Advantages and Drawbacks. APT Bull. J. Preserv. Technol..

[64] Hafez I.T., Biskos G. (2021). New Method for the Protection and Restoration of Calcareous Cultural Heritage Stones by Polyelectrolytes and Hydroxyapatite Nanocrystals. J. Colloid Interface Sci..

[65] Manoudis P., Papadopoulou S., Karapanagiotis I., Tsakalof A., Zuburtikudis I., Panayiotou C. (2007). Polymer-Silica Nanoparticles Composite Films as Protective Coatings for Stone-Based Monuments. J. Phys. Conf. Ser..

[66] Chobba M.B., Weththimuni M.L., Messaoud M., Sacchi D., Bouaziz J., De Leo F., Urzi C., Licchelli M. (2021). Multifunctional and Durable Coatings for Stone Protection Based on Gd-Doped Nanocomposites. Sustainability.

[67] Andreotti S., Franzoni E., Ruiz-Agudo E., Scherer G.W., Fabbri P., Sassoni E., Rodriguez-Navarro C. (2019). New Polymer-Based Treatments for the Prevention of Damage by Salt Crystallization in Stone. Mater. Struct..

[68] Rinaudo M., Belgacem M.N., Gandini A. (2008). Polyelectrolytes Derived from Natural Polysaccharides. Monomers, Polymers and Composites from Renewable Resources.

[69] Lettieri M., Masieri M., Pipoli M., Morelli A., Frigione M. (2019). Anti-Graffiti Behavior of Oleo/Hydrophobic Nano-Filled Coatings Applied on Natural Stone Materials. Coatings.

[70] Zárraga R., Cervantes J., Salazar-Hernandez C., Wheeler G. (2010). Effect of the Addition of Hydroxyl-Terminated Polydimethylsiloxane to TEOS-Based Stone Consolidants. J. Cult. Herit..

[71] Kapridaki C., Pinho L., Mosquera M.J., Maravelaki-Kalaitzaki P. (2014). Producing Photoactive, Transparent and Hydrophobic SiO_2_-Crystalline TiO_2_ Nanocomposites at Ambient Conditions with Application as Self-Cleaning Coatings. Appl. Catal. B Environ..

[72] Kapridaki C., Verganelaki A., Dimitriadou P., Maravelaki-Kalaitzaki P. (2018). Conservation of Monuments by a Three-Layered Compatible Treatment of TEOS-Nano-Calcium Oxalate Consolidant and TEOS-PDMS-TiO2 Hydrophobic/Photoactive Hybrid Nanomaterials. Materials.

[73] La Russa M.F., Rovella N., Alvarez de Buergo M., Belfiore C.M., Pezzino A., Crisci G.M., Ruffolo S.A. (2016). Nano-TiO_2_ Coatings for Cultural Heritage Protection: The Role of the Binder on Hydrophobic and Self-Cleaning Efficacy. Prog. Org. Coat..

[74] Crupi V., Fazio B., Gessini A., Kis Z., La Russa M.F., Majolino D., Masciovecchio C., Ricca M., Rossi B., Ruffolo S.A. (2018). TiO_2_–SiO_2_–PDMS Nanocomposite Coating with Self-Cleaning Effect for Stone Material: Finding the Optimal Amount of TiO_2_. Constr. Build. Mater..

[75] Aricov L., Băran A., Simion E.L., Gîfu I.C., Anghel D.-F., Jerca V.V., Vuluga D.M. (2016). New Insights into the Self-Assembling of Some Hydrophobically Modified Polyacrylates in Aqueous Solution. Colloid Polym. Sci..

[76] Aricov L., Petkova H., Arabadzhieva D., Iovescu A., Mileva E., Khristov K., Stinga G., Mihailescu C.-F., Anghel D.F., Todorov R. (2016). Aqueous Solutions of Associative Poly(Acrylates): Bulk and Interfacial Properties. Colloids Surf. Physicochem. Eng. Asp..

[77] Aricov L., Băran A., Stîngă G., Simion E.L., Gîfu I.C., Anghel D.-F., Rădiţoiu V. (2017). Formation and Hosting Properties of Polyacrylate–Surfactant Complexes. Colloid Polym. Sci..

[78] Gîfu I.C., Maxim M.E., Cinteza L.O., Popa M., Aricov L., Leontieș A.R., Anastasescu M., Anghel D.-F., Ianchis R., Ninciuleanu C.M. (2019). Antimicrobial Activities of Hydrophobically Modified Poly(Acrylate) Films and Their Complexes with Different Chain Length Cationic Surfactants. Coatings.

[79] Gîfu I.C., Maxim M.E., Iovescu A., Simion E.L., Aricov L., Anastasescu M., Munteanu C., Anghel D.-F. (2016). Surface Hydrophobization by Electrostatic Deposition of Hydrophobically Modified Poly(Acrylates) and Their Complexes with Surfactants. Appl. Surf. Sci..

[80] Gîfu I.C., Maxim M.E., Iovescu A., Aricov L., Simion E.L., Leontieş A.R., Anastasescu M., Munteanu C., Anghel D.-F. (2017). Natural Aging of Multilayer Films Containing Hydrophobically Modified Poly(Acrylate)s or Their Complexes with Surfactants. Appl. Surf. Sci..

[81] Fruth V., Todan L., Codrea C.I., Poenaru I., Petrescu S., Aricov L., Ciobanu M., Jecu L., Ion R.M., Predoana L. (2021). Multifunctional Composite Coatings Based on Photoactive Metal-Oxide Nanopowders (MgO/TiO_2_) in Hydrophobic Polymer Matrix for Stone Heritage Conservation. Nanomaterials.

[82] Lo Schiavo S., De Leo F., Urzì C. (2020). Present and Future Perspectives for Biocides and Antifouling Products for Stone-Built Cultural Heritage: Ionic Liquids as a Challenging Alternative. Appl. Sci..

[83] Wu T., Yang Y., Su H., Gu Y., Ma Q., Zhang Y. (2022). Recent Developments in Antibacterial or Antibiofilm Compound Coating for Biliary Stents. Colloids Surf. B Biointerfaces.

[84] Li Q., Wu C., Zhang B. (2022). Hybrid Hydrogels Based on Polyvinyl Alcohol, Branched Polyethylenimine, Polydopamine, and Phosphonium-Based Ionic Liquid for Effective Synergetic Antibacterial Applications. Colloids Surf. Physicochem. Eng. Asp..

[85] Kanth A.P., Soni A.K. (2023). Application of Nanocomposites for Conservation of Materials of Cultural Heritage. J. Cult. Herit..

[86] Youssef A.M., Kamel S., El-Samahy M.A. (2013). Morphological and Antibacterial Properties of Modified Paper by PS Nanocomposites for Packaging Applications. Carbohydr. Polym..

[87] Romani M., Warscheid T., Nicole L., Marcon L., Di Martino P., Suzuki M.T., Lebaron P., Lami R. (2022). Current and Future Chemical Treatments to Fight Biodeterioration of Outdoor Building Materials and Associated Biofilms: Moving Away from Ecotoxic and towards Efficient, Sustainable Solutions. Sci. Total Environ..

[88] Pinna D. (2022). Can We Do without Biocides to Cope with Biofilms and Lichens on Stone Heritage?. Int. Biodeterior. Biodegrad..

[89] Sfameni S., Rando G., Plutino M.R. (2023). Sustainable Secondary-Raw Materials, Natural Substances and Eco-Friendly Nanomaterial-Based Approaches for Improved Surface Performances: An Overview of What They Are and How They Work. Int. J. Mol. Sci..

[90] Ruggiero L., Bartoli F., Fidanza M.R., Zurlo F., Marconi E., Gasperi T., Tuti S., Crociani L., Di Bartolomeo E., Caneva G. (2020). Encapsulation of environmentally-friendly biocides in silica nanosystems for multifunctional coatings. Appl. Surf. Sci..

[91] Liu Y., Suo X., Wang Z., Gong Y., Wang X., Li H. (2017). Developing Polyimide-Copper Antifouling Coatings with Capsule Structures for Sustainable Release of Copper. Mater. Des..

[92] Belgacem M.N., Gandini A. (2011). Monomers, Polymers and Composites from Renewable Resources.

[93] Ravi Kumar M.N.V. (2000). A Review of Chitin and Chitosan Applications. React. Funct. Polym..

[94] Giuliani C., Pascucci M., Riccucci C., Messina E., Salzano de Luna M., Lavorgna M., Ingo G.M., Di Carlo G. (2018). Chitosan-Based Coatings for Corrosion Protection of Copper-Based Alloys: A Promising More Sustainable Approach for Cultural Heritage Applications. Prog. Org. Coat..

[95] Zhou S., Zhao Z., Mao H., Wang L., Chen J., Chen J., Huang X. (2022). Bronze Preservation by Using Composite Hydrogel Coating-Loaded Corrosion Inhibitors. Herit. Sci..

[96] Zheludkevich M.L., Shchukin D.G., Yasakau K.A., Möhwald H., Ferreira M.G.S. (2007). Anticorrosion Coatings with Self-Healing Effect Based on Nanocontainers Impregnated with Corrosion Inhibitor. Chem. Mater..

[97] Abu-Thabit N.Y., Hamdy A.S. (2016). Stimuli-Responsive Polyelectrolyte Multilayers for Fabrication of Self-Healing Coatings—A Review. Surf. Coat. Technol..

[98] Wandrey C. (2005). Polyelectrolytes. Polym. News.

[99] Andreeva D.V., Skorb E.V., Shchukin D.G. (2010). Layer-by-Layer Polyelectrolyte/Inhibitor Nanostructures for Metal Corrosion Protection. ACS Appl. Mater. Interfaces.

[100] Ntelia E., Karapanagiotis I. (2020). Superhydrophobic Paraloid B72. Prog. Org. Coat..

[101] Quintero Balbas D., Dal Fovo A., Porcu D., Chaban A., Porcinai S., Fontana R., Striova J. (2022). Non-Invasive Evaluation of Polymeric Protective Coatings for Metal Surfaces of Cultural Heritage Objects: Comparison of Optical and Electromagnetic Methods. Appl. Sci..

[102] Sadat-Shojai M., Ershad-Langroudi A. (2009). Polymeric Coatings for Protection of Historic Monuments: Opportunities and Challenges. J. Appl. Polym. Sci..

[103] Trovato V., Rosace G., Colleoni C., Sfameni S., Migani V., Plutino M.R. (2020). Sol-Gel Based Coatings for the Protection of Cultural Heritage Textiles. IOP Conf. Ser. Mater. Sci. Eng..

[104] Baglioni P., Chelazzi D., Giorgi R., Poggi G. (2013). Colloid and Materials Science for the Conservation of Cultural Heritage: Cleaning, Consolidation, and Deacidification. Langmuir.

[105] D’Orazio L., Gentile G., Mancarella C., Martuscelli E., Massa V. (2001). Water-Dispersed Polymers for the Conservation and Restoration of Cultural Heritage: A Molecular, Thermal, Structural and Mechanical Characterisation. Polym. Test..

[106] Mazzon G., Zanocco I., Zahid M., Bayer I., Athanassiou A., Falchi L., Balliana E., Zendri E. (2017). Nanostructured Coatings for the Protection of Textiles and Paper. Ge-Conservacion.

[107] Jia M., Zhang X., Weng J., Zhang J., Zhang M. (2019). Protective Coating of Paper Works: ZnO/Cellulose Nanocrystal Composites and Analytical Characterization. J. Cult. Herit..

[108] Jia Z., Yang C., Zhao F., Chao X., Li Y., Xing H. (2020). One-Step Reinforcement and Deacidification of Paper Documents: Application of Lewis Base—Chitosan Nanoparticle Coatings and Analytical Characterization. Coatings.

[109] Castillo I.F., De Matteis L., Marquina C., Guillén E.G., Martínez de la Fuente J., Mitchell S.G. (2019). Protection of 18th Century Paper Using Antimicrobial Nano-Magnesium Oxide. Int. Biodeterior. Biodegrad..

[110] Chollakup R., Kongtud W., Sukatta U., Piriyasatits K., Premchookiat M., Jarerat A. (2020). Development of Rice Straw Paper Coated with Pomelo Peel Extract for Bio-Based and Antibacterial Packaging. Key Eng. Mater..

[111] Spagnuolo L., D’Orsi R., Operamolla A. (2022). Nanocellulose for Paper and Textile Coating: The Importance of Surface Chemistry. ChemPlusChem.

[112] Zhang J., Zhang D., Zhang X. (2020). UV-0/HPC Laminated Coatings for Protection of Cellulosed-Based Cultural Heritage against UV Rays. Polym. Degrad. Stab..

[113] Totolin M.I., Neamţu I. (2011). Positive Findings for Plasma Polymer (Meth)Acrylate Thin Films in Heritage Protective Applications. J. Cult. Herit..

[114] Zhou H., Wang H., Niu H., Fang J., Zhao Y., Lin T. (2015). Superstrong, Chemically Stable, Superamphiphobic Fabrics from Particle-Free Polymer Coatings. Adv. Mater. Interfaces.

